# Beyond reverse pharmacology: Mechanism-based screening of Ayurvedic drugs

**DOI:** 10.4103/0975-9476.74435

**Published:** 2010

**Authors:** R. D. Lele

**Affiliations:** *Lilavati Hospital and Research Center, Mumbai, India*

**Keywords:** Ayurveda, Reverse Pharmacology

## Abstract

This paper reviews the pharmacology of Indian medicinal plants, starting with the historical background of European work on the subject beginning as early as the 17th century, and tracing its history through the work of Sen and Bose in the 1930‘s, and Vakhil’s historic 1949 paper on Sarpaghanda. The often crucial role of patient feedback in early discoveries is highlighted, as is the time lag between proof of pharmacological action and identification of the active principle, and subsequent elucidation of mechanism of action. In the case of Indian plants in the 20th century this process sometimes took almost 50 years. Reserpine and its mechanisms are given in detail, and its current relevance to public health discussed. The foundation of present day methods of pharmacology is briefly presented so the complexity of methods used to identify properties of Ayurveda derived drugs like forskolin and baicalein, and their bioavailability, may be better appreciated. Ayurveda derived anti-oxidants and their levels of action, immuno-modulators, particularly with respect to the NF-kB pathway and its implications for cancer control, are all considered. The example of curcumin derived from turmeric is explained in more detail, because of its role in cancer prevention. Finally, the paper emphasizes the importance of Ayurveda’s concepts of rasayana as a form of dietary chemo-prevention; the significance of ahar, diet, in Ayurveda’s aspiration to prevent disease and restore health thus becomes clear. Understood in this light, Ayurveda may transcend pharmacology as a treatment paradigm.

## INTRODUCTION

The most important single event that aroused the interest of modern medicine in the Ayurvedic Pharmacopoeia was Dr. Rustum Jal Vakil’s 1949 report in the *British Heart Journal* on the usefulness of serpina (whole extract of Sarpagandha – *Rauwolfia serpentina*) in the treatment of hypertension.[1] Its pharmacological properties had been investigated earlier by Sen and Bose in 1931 and Paranjpe in 1942. Sen and Bose not only demonstrated it to have antihypertensive effects but also noted certain side effects such as depression, parkinsonism, gynecomastia, dyspeptic symptoms and so on.

Sen and Bose were using an approach similar to reverse pharmacology which had earlier been used by European physicians for three centuries since the Renaissance, gaining from the experience of shamans, witch doctors and religious preachers. Thomas Sydenham (1624-1689) used quinine to separate malaria from other fevers, and colchicine to separate gout from other arthritis; Edward Stone (1763) used willow bark decoction for "cure of agues" expecting that it would cure malaria, but it helped in treating rheumatic fever and not malaria; William Withering (1775) used foxglove to treat dropsy, getting a clue from a Shropshire lady suffering from it; in 1914, Wenchebach, the Dutch physician, used quinine and quinidine for cardiac arrhythmias after getting a clue from a Dutch sailor who told him “when I take quinine my palpitations disappear!”

Table 1 lists examples of plant-derived drugs used in modern medicine. It is noteworthy that knowledge about their mechanism of action at the molecular level has come only in the last three decades.

**Table 1:** Plant origin of drugs used in modern medicine

Drug	Plant source	Clinical observation	Molecular mechanism of action
Artemether	Qinshausu	Chloroquin resistant malaria	Heme–mediated decomposition of endoperoxide, generating free radicals
Atropin	*Atropa belladonna*	Antispasmodic	MAch receptors
Caffeine	*Coffee Arabica*	Stimulant	Adenosine receptors
*Cannabis indica*	Indian Hemp	Sedation, antiemetic	Cannabinoid receptors
Cocaine	Leaves of Coca	Addictive drug	CB_1_ CB_2_
Colchicine	*Colchicum autumnale*	Relief of pain in gout	Blocks DAT, NET, SERT Inhibits release of leukocyte-derived chemotactic factors
Digitalis	Foxglove	Relief of dropsy	Na+K+ATPase
Emetine	Ipecacuana	Amoebic dysentery	Inhibits protein synthesis in eukaryotic cells
Ephedrine	Ephedra	Bronchodilator	α, β adrenoreceptor agonist
Eserine	Calabar beans	Pupil constriction	Reversible cholinesterase inhibitor
Morphine	*Papavarum somniferum*	Analgesic	Opioid receptors
Nicotine	Tobacco plant	Stimulant	Nicotinic Ach receptors
Quinine	Cinchona bark	Fever due to malaria	Inhibits hemozoin crystallization – aggregation of cytotoxic heme
Reserpine	Sarpagandha	Sedation, lowers BP	Blocks VMAT_1,_ VMAT_2_
Salicylic acid	*Salix alba* Willow bark	Fever and pain relief	Cox inhibitor NFKB inhibitor
Strychnine	*Nux vomica*	Hyperexcitability convulsions	Blocks glycine receptors
Vincristine	*Vinca rosea*	Anticancer	Binds to tubulin disrupts microtubule assembly

## RESERPINE – A UNIQUE MOLECULE

The active principle of Sarpagandha, reserpine, was identified in 1978. Transporters for the three biogenic amines – norepinephrine (NE), dopamine and serotonin – were discovered in the 1990s. Abbreviated NET, DAT and SERT, respectively, these transporters are of particular clinical interest because they are the molecular target for many antidepressants, as well as drugs of abuse such as cocaine and amphetamine. The vesicular monoamine transporters (VMATs) were discovered in 1998 and their distribution found: VMAT2 localized to neuronal tissue, while VMAT1 localized in endocrine tissues. Vesicular transporters play major roles in packaging neurotransmitters into distinct secretory vesicles in preparation for subsequent exocytotic release, thus controlling the quantal size of each release.

Reserpine is a unique molecule in that it blocks both VMAT1 and VMAT2, thereby exposing biogenic amines to degradation by monoamine oxidase (MAO). Even today, it remains unique. Another molecule, tetrahydrobenazine, inhibits VMAT2 but not VMAT1.

### Relevance of reserpine in the 21st century

According to Braunwald (Heart 6th ed. 2001), “reserpine reduces blood pressure by inhibiting vesicular uptake of NE in post ganglionic adrenergic neurons, thereby exposing it to degradation by cytoplasmic MAO. Peripheral effects are predominant, although central NE stores are also depleted leading to sedation and depression. Single daily oral doses of 0.05 mg (as effective as higher doses 0.125 or 0.25 mg) make reserpine the most effective inexpensive single drug for the control of hypertension, but it is ignored as it has no commercial sponsor”.

Dr. M. K. Mani has been using reserpine plus the diuretic thiazide as a single daily dose to control hypertension in village populations in Chennai for the last 5 years. As regards fear of depression as a side effect, Mani says, “Millions of Indian villagers are already living in such miserable and depressing conditions that 0.05 mg of reserpine is not going to make them worse, especially if they understand that it will protect them from chronic renal failure over the next two decades”.

Two questions to be urgently answered are: (1) Does the standardized watery extract (sarpagandha ghanavati) cause less depression and extra pyramidal side effects than pure reserpine, while achieving the same success in lowering blood pressure? and (2) How much extract is required to be given orally to match 0.05 mg reserpine?

Ayurvedic drug companies have a great opportunity to extend the benefit of this unique Ayurvedic molecule as the most cost-effective solution for millions of hypertensives in India and the developing world.

(Fear of depression due to reserpine reminds me of another fear – lactic acidosis – with the routine use of metformin for type 2 diabetes. Indian physicians have been using metformin for over 40 years without encountering this problem, the fear of which kept American physicians deprived of this drug for three decades till 1995.)

## MOLECULAR BIOLOGY AND MOLECULAR MEDICINE: 21ST CENTURY PARADIGM

The union of biology with physics, chemistry, mathematics and computer science was an outstanding development of the 20th century science. Physical and chemical approaches to problems in biology became increasingly productive, giving rise to new concepts in molecular biology and molecular medicine. The confluence of several powerful methods of observation – chemical analysis, electron microscopy, X-ray crystallography, electron spin resonance (ESR), and nuclear magnetic resonance (NMR) spectroscopy - eventually led to the determination of the precise double helix architecture of DNA, three-dimensional configurations of protein molecules and amino acid sequences of their constituent polypeptide chains, and the precise characterization and three-dimensional structure of most biologically active molecules. The synthesis of complex lipids and carbohydrates, the function of cell membranes and partitioning of inorganic ions occur as a secondary consequence of the action of specific proteins. Many of these proteins are enzymes that catalyze the biochemical conversion of one molecule into another. Some are structural proteins such as collagen or elastin; others are regulatory proteins that dictate how much of each enzyme or each structural protein is made, when and where. All this new knowledge can be considered an elaboration of the Ayurvedic concept of “Rasa Dhatu” and should be eagerly assimilated by Ayurvedic physicians following the exhortations of Charaka, Sushrut and Vagbhat.

The human body is made up of trillions of cells which constantly communicate with each other through recognition molecules. Molecular recognition is the fundamental feature of all biological processes encompassing ligand–receptor, substrate–enzyme, and antigen–antibody reactions. Nuclear Medicine is uniquely placed to study biological processes at the molecular level. Molecular Nuclear Medicine offers the opportunity to study physiology and biochemistry at the molecular level in the living human body including the brain. Radiochemists have developed stereospecific ligands with the proper charge, shape and lipophilicity for transport across cell membranes or the blood brain barrier.

The RBI Hand Book of Receptor Classification and Signal Transduction gives a detailed list of over 80 classes of receptors and transporters, intracellular signaling molecules and ion channels, classified into component subtypes and subgroups, structure, selective agonists, selective antagonists, signal transduction mechanisms, tissue distribution and specific radioligands of choice [Table 2]. This resource should be fully utilized for mechanism-based screening of Ayurvedic herbal drugs.

**Table 2:** List of receptors and transporters for which specific radiolabeled ligands are available for drug screening

Non peptide	Peptide
Acetyl choline receptors	Angiotensin
Muscarinic M_1_, M_2_, M_3_, M_4_, M_5_	AT_1_ AT_2_
Nicotinic four types	Bombesin
Adenosine	BB_1_ BB_2_, BB_3_
A_1_, A_2_,A, A_2_B, A_3_	Bradykinin
Adrenoceptors	B_1_, B_2_
α_1_A, α_2_B, α_1_∆, α_2_A, α_2_B, α_2_X;,	Calcitonin-gene related peptide
α2∆	receptor
β_1_, β_2_, β_3_	CGRP_1_, _2_, amylin, adrenomedullin
Biogenic amine transporters	Chemokine
NET, DAT, SERT	CXCR_1_, _2_, _3_, _4_
Cannabinoid	CCR_4_, _5_, _6_, _7_, _8_, _9_, _10_, _11_
CB_1_, CB_2_	XCRI, CX3CRI, DARC
Dopamine	ECRF_3_, US_2_8, KSHV
D_1_, D_2_, D_3_, D_4_, D_5_	Cholecystokinin/gastrin
GABA receptors	CCKA, CCKB
A, B, C	Corticotropin releasing factor
GABA transporters	CRF_1_, CRF_2α, 2β, 2γ_, CRF-BP
GAT-_11, 2, 3,_ BGT, VGAT	Cytokine
Excitatory amoinoacid transporters	Hematoprotein family
EAAT_1_, T_2_, T_3_, T_4_, T_5_	Il-_2, 3, 4, 5, 6, 7, 8, 9, 10, 11,_
Glutamate	_12, 12, 15, 16, 17, 19, 21, 22_
G protein family: eight types	Tumor necrosis family 9
Ion channel family: three types	ILIR/TIR
Glycinic receptor	IL1R1, IL1RII, IL-18
GlyT1, GlyT2	TNF receptor family
Histamine	TNFRSF _1, 2, 3, 4, 5, 6, 7, 8, 9,_
H_1_, H_2_, H_3_, H_4_	_10_A, B, C, D, _11_
Imidazoline binding sites	Endothelin
I_1_, I_2_, I_3_	ETA ETB
Leukotriene	Galanin
BLT_1_, BLT_2_, CysLT, CysLT_2_	R1 R2 R3
Lysophospholipid	Melanocortin
P_1_, P_2_, P_3_, P_4_, P_5_	MCR1, 2, 3, 4, 5, 6
Melanin concentrating hormone	Neuropeptidase
MCH_1_, MCH_2_	Neuropeptide
Melatonin	Y_1_ Y_2_ Y_4_ Y_5_ Y_6_
MT_1_, MT_2_, MT_3_	Neurotensin
Platelet activating factor receptor	NT_1_ NT_2_
Prostanoid	Neurotrophin
EP_1_, EP_2_, EP_3_, EP_4_	TrK A, B, C, p75
P_2_ P_2_X subtype (ion channel	Opiod receptors
family) 7	
P_2_Y subtype (G protein family) 7	δ (OP1) k(OP2) μ(OP3), OP4
Serotonin	Orexin receptors
5HT_1_A 5HT_1_B, 5HT_1_D SHT_1_f	O×1 O×2
5HT_2_, 5HT_1_D 5HT_2c_ 5HT_3_ 5HT_4_	Proteinase-activated
5HT_5_ 5HT 5HT_6_ 5HT_7_	PAR1, 2, 3
Ion channels:	Somatostatin
Calcium channels	SST _1, 2, 3, 4,5_
Chloride channels	Tachykinin
Potassium channels	NK1, NK2, NK3
Sodium channels	VIP
Sigma receptor	VPAC_11, 2,_ PAC_1_
Vanilloid receptors	Vasopressin and oxytocin
	receptor
V_1a_, V_1b_, V_2_, OT	VEGF
	_1, 2, 3_
Intracellular signaling enzymes/receptors	
Adenylyl cyclases – 10 isoenzymes	
Ca+ calmodulin dependent protein kinases	
Caspases	
Cyclic nucleotide-regulated kinases	
Cyclic nucleotide phosphodiesterases	
Cyclin-dependent kinases	
G-protein coupled receptor kinases	
Heteromeric G proteins	
Ins P_3_/Ryanodine receptors	
Intracellular receptors (non-steroid) (steroid)	
Mitogen-activated protein kinase	
Nitric oxide synthases	
PDK1-PKB/AKT signaling	
Peroxisome proliferators activated receptor (PPARs)	
Phosphoinositide kinases	
Phospholipase A_2_	
Phospholipase C (phosphoinositide specific)	
Phospholipase D (phosphatidylcholine specific)	
Phosphoprotein phosphatases	
Serine/threonine phosphatases	
Protein tyrosine phosphatases	
Protein kinase C	
Protein prenyl transferases	
Small molecular weight G proteins	
Tyrosine kinases	
Receptor linked	
Non-receptor linked	
Ion channel	
Calcium: L, T, N, P, Q, R	
Chloride: CIC, CFTR, GABA/glycine	
Potassium: KIR, ATP-sensitive, Tandem pore, voltage-gated Ca2+	
activated, KCNQ, HERG	
Sodium: I, II, IIA, III, _μ1_, PN_1_, V_1_, h_1_, PN_3_βNS, SNS_2_	
Vanilloid receptors (capsicum – capsaicin) activated	

## FORSKOLIN, A UNIQUE AYURVEDIC COMPOUND

Sir Ramnath Chopra (1882–1973) was a pioneer in the field of experimental pharmacology of indigenous Indian drugs, in evaluating the effects of Ayurvedic drugs and plant extracts on tissues and animals. Today, with the use of radiotracers and nuclear imaging techniques, we propose to break new grounds in understanding the action of Ayurvedic drugs at the molecular level, particularly the *Rasayana* drugs and *Medhya Rasayanas* (memory enhancing drugs).

That the possibility of discovering new gems from Ayurveda is by no means exhausted is shown by another discovery: that of Forskolin, the active principle in the Indian plant *Coleus forskohlii* Brig (Sanskrit name: Gandira; Marathi name: Mainmool), mentioned in Nadkarni’s Materia Medica (1908). In the 1970s, scientists from Hoechst Pharmaceuticals showed Forskolin to be a unique diterpene, which activates cell membrane bound adenylate cyclase and cAMP, increasing cardiac contractility. Using the Nuclear Stethoscope to continuously measure systolic and diastolic function in the living human heart, I studied the effect of IV infusion of Forskolin in 30 patients of congestive heart failure. Ventricular systole (inotropic function) and diastole (lusitropic function) are both active processes - diastole is not a passive relaxation, but an active process requiring cAMP. It was observed that Forskolin improved both systolic and diastolic function at constant preload and after load. Nitroglycerine decreases preload and after load, thereby improving systolic and diastolic function. Hence, it was established that Forskolin is inotropic and lusitropic, unlike digoxin which is only inotropic. Unfortunately, like reserpine, Forskolin is not available as an oral drug.

## ARJUNA – “CARDIAC TONIC”, “CARDIOPROTECTIVE”

When BHU Prof. Deshpande of “Ksharasutra” fame learned about my study of Forskolin in 1983, he urged me to study Arjuna, which Ayurveda describes as a “cardiac tonic”. With the Nuclear Stethoscope, I designed a study similar to the one with Forskolin, but did not find any inotropic or lusitropic function improvement. Hence, in contrast to Forskolin, Arjuna was not cardiotonic. So what other mechanism could be operative? High degrees of oxidative stress occur in cardiovascular disease, especially in association with diabetes and hypertension. However, Gupta *et al*, (2001) found antioxidant and hypocholesterolemic effects in *Terminalia arjuna* bark powder in a randomized placebo-controlled trial.[4] So, could Arjuna be “cardioprotective” through antioxidant effects? To prove this hypothesis, Devasagayam and his group at BARC studied the reaction of *T. arjuna* extracts and its active principle, baicalein, with the biologically important superoxide (O_2_^–^) and singlet oxygen (_1_O_2_) by measuring O_2_^–^and O_2_ induced damage of lipids by lipid peroxidation in reaction mixtures containing rat liver mitochondria, and cardiac homogenate. Even at low concentration of 21–25 μM, baicalein was shown to be highly effective in inhibiting lipid peroxidation. Further, both *T. arjuna* extracts and baicalein were shown to possess higher scavenging activities than standard antioxidants. This was confirmed by different physicochemical methods such as spin trapping by ESR and pulse radiolysis.[2,3]

Blood samples taken from normal healthy non-smoking volunteers were obtained to study the protective effects of different concentrations of baicalein against plasma oxidation in the presence of 2.2-azobis(2-amindinopropane) dihydrochloride (AAPH) at 37°C. Spectroscopic measurements indicate lipoprotein oxidation induced *in vitro* in the whole plasma. 50 μM baicalein was the most effective and showed higher protection than that of 50 μM trolox, a standard antioxidant. In the case of *T Arjuna* extracts, 50 μg/ml aqueous extract was better than methanolic extract in decreasing plasma lipid peroxidation.

Baicalein inhibits the binding of a number of chemokines to human leukocytes, reducing their migration capacity. The action is selective to CXC, CC (MIP-1β) and MCP-1, thus controlling the inflammatory response.[18] This has great relevance to the prevention of atherosclerosis.

Baicalein inhibits fibrillation of α synuclein and disaggregates existing fibrils in neurons. Aggregation of α synuclein is a critical step in the development of Parkinson’s disease, hence baicalein may have a prevention role.[17]

### Bioavailability study of Arjuna

Bioavailability of Ayurvedic herbal drugs is a totally neglected subject. Hence, intestinal absorption of *T. arjuna* extracts and baicalein was studied by Devasagayam’s group using inverted loop of rat intestine, with the absorbed components monitored by high performance liquid chromatography (HPLC). Almost 25% of baicalein (4 mg/ml) was recovered from the serosal surface. Both aqueous and methanolic extracts of *T. arjuna* (1 mg/ml) were absorbed. It was suggested that the rat inverted loop technique should be routinely used to establish bioavailability of Ayurvedic herbal drugs. If a concentration of 50 μg/ml is found effective *in vitro*, then the amount of drug to be taken orally required to achieve this concentration will only be answered by bioavailability studies. This “blind spot” in Ayurveda herbal drug research is both amazing and frustrating for clinicians wishing to translate *in vitro* laboratory data into clinical applications. Poor bioavailability of oral curcumin and resveratrol are important illustrative examples.

## AYURVEDIC ANTIOXIDANTS

**Figure 1:**
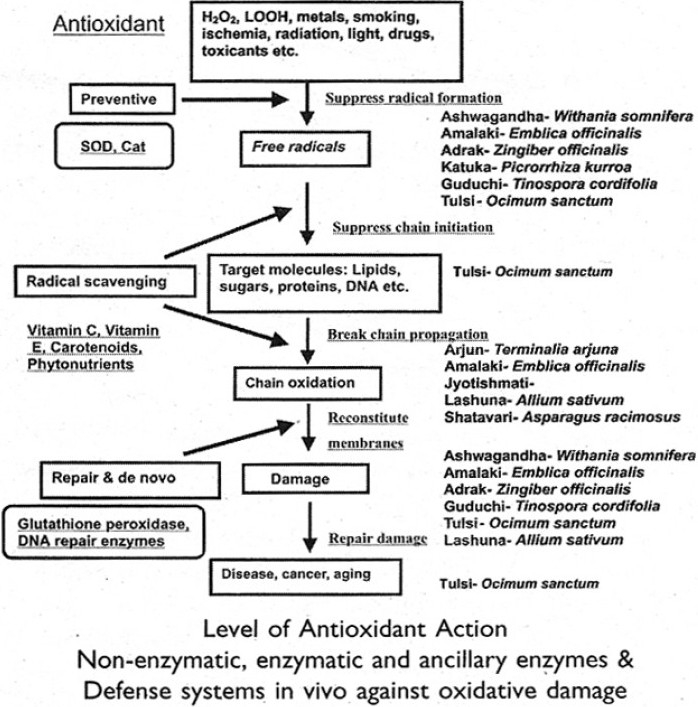
Ayurvedic anti-oxidants

Devasagayam’s group at BARC have studied Ayurvedic antioxidants according to five levels of action, viz., suppress radical formation, break chain initiation, break chain propagation, reconstitute membrane and repair damage, spelling out methods to be used according to level of action [Figure 1].

Antioxidant capacity assays may be broadly classified as electron transfer (ET) and hydrogen atom transfer (HAT) based assays. The majority of HAT assays are kinetics based and involve a competitive reaction scheme, in which antioxidant and substrate compete for peroxyl radicals thermally generated through decomposition of azo compounds. ET based assays measure the capacity of an antioxidant in the reduction of an oxidant, which changes color when reduced. ET assays include the trolox equivalent antioxidant capacity (ABT/TEAC), Modified cupric reducing antioxidant capacity (CUPRAC), 2,2-diphenyl-1-picrylhydrazyl (DPPH), ferric reducing ability of plasma (Folin-Ciocalteu and (FRAP ferric reducing ability of plasma) methods, each using different chromogenic redox reagents with different standard potentials [Table 3].

## AYURVEDIC IMMUNOMODULATORS

Dahanukar and Thatte studied six Ayurveda *Rasayanas* selected because they were specified as *ekadravyas*, i.e., they can be given as single entities:

Amla: *Emblica officinalis* (EO)

Ashwagnadha: *Withania somnifera* (WS)

Guduchi: *Tinospora cordifolia*[13] (TC)

Haritaki: *Terminalia chebula* (TCh)

Pippali: *Piper longum* (PL)

Shatavari: *Asparagus racemosus* (AR).[15]

A dose of 100 mg/kg was selected to be given orally as total aqueous extracts for 1-2 weeks. They showed that the aqueous extracts of Guduchi stimulated phagocytic and bactericidal activity of neutrophils and macrophages. Pre-treatment with all six Rasayanas was effective in protecting the animals from infection to varying degrees.

**Figure 2:**
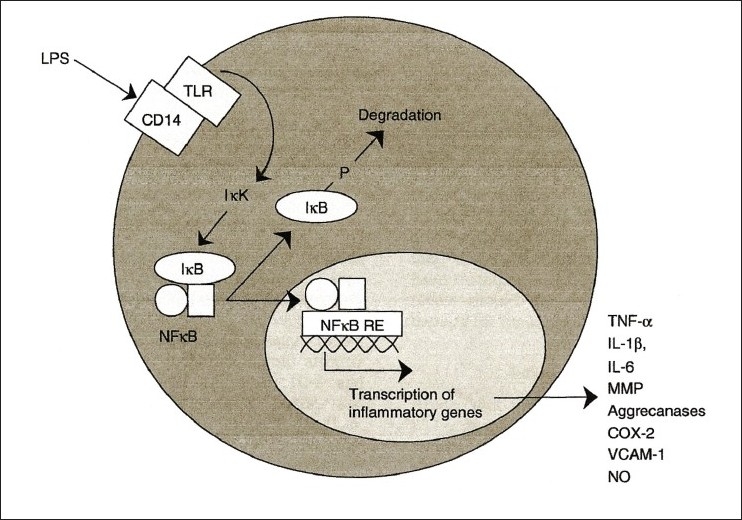
Role of NFKB as regulator of inflammatory response

**Figure 3:**
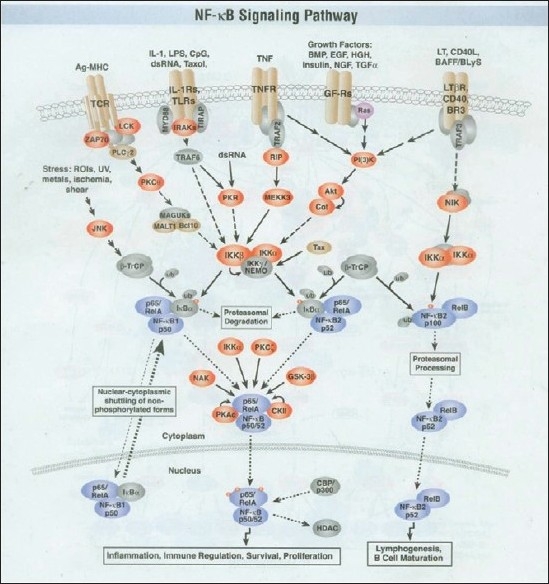
The NFKB signaling pathway

**Figure 4:**
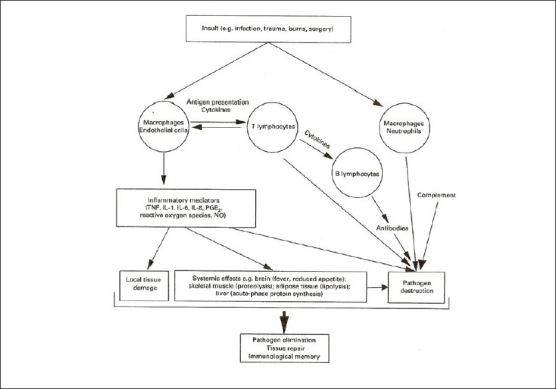
The Interrelationship between components of the innate and acquired immune response. It interleukin, PG, prostaglandin, TNF, tumour necrosis factor

At that time, not much was known about the key role of NF-kB (nuclear factor kappa-light-chain-enhancer of activated B cells) as a regulator of host inflammatory and immune response and cellular growth [Figures 2 and 3]. Agarwal and Singh’s excellent review of Indian medicinal plants as immunomodulators (1999) makes no mention of the NF-kB pathway.[8] NF-kB increases the expression of specific cellular genes encoding at least 27 different cytokines and chemokines, receptors involved in immune recognition such as members of the major histocompatibility complex (MHC) proteins involved in antigen presentation, and receptors required for neutrophil adhesion and migration. Cytokines stimulated by NF-kB, such as interleukin (IL)-1β and tumor necrosis factor (TNF)α, also directly activate NF-kB pathways, thus establishing a positive autoregulatory loop amplifying the inflammatory response and increasing the duration of chronic inflammation.[10] NF-kB also stimulates the expression of enzymes whose products contribute to the pathogenesis of the inflammatory process, e.g., inducible nitric oxide synthase (iNOS), which generates nitric oxide (NO), and cyclooxygenase - 2 (COX-2), which generates prostanoids [Figure 4].

NF-kB controls immune response by modulation of B lymphocyte survival, mitogen dependent cell proliferation and isotype switching, which lead to the differentiation of B lymphocytes, IL-2 production which increases proliferation and differentiation of T lymphocytes.

IkB protein normally binds to NF-kB, thereby blocking nuclear translocation. Lipopolysaccharide (LPS), phorbol esters, viral infections, ultraviolet radiation and free radicals all lead to degradation of IkB, thereby releasing nuclear translocation of NF-kB. NF-kB proteins possess site and event specificity. Tissue distribution and time duration differ for various IkBs. IkBα is associated with transient NF-kB activation while IkBβ is associated with sustained NF-kB activation.

Interestingly, NF-kB activation is also a key pathway for carcinogenesis.

The role of NF-kB activation in human cancer is suggested by increased NF-kB levels in the nuclei of several types of cancer such as leukemia, lymphoma, solid tumors of breast, ovary, prostate and colon cancer. Possibly, mutations inactivating IkB protein activate NF-kB pathways. Equally, NF-kB pathway inhibition may enhance the efficacy of cancer chemotherapy.

Better understanding of NF-kB pathway regulation provides opportunities to develop treatments to inhibit prolonged activation of the pathway, especially in conditions which become chronic or dysregulated.[9] The pioneering work of Dahanukar and Thatte has shown that Ayurvedic *Rasayanas* can re-regulate a chronic or dysregulated NF-kB pathway.

### Molecular mechanisms of herbal immunomodulators

Sainis and his group at BARC (1999) isolated a high molecular weight polysaccharide, arabinogalacton, from Guduchi dry stem crude extract (DSCE) – G1-4A (Indian patent No. 56/ Bom. 98) with immunostimulant action on polymorphonuclear (PMN) leukocytes and macrophages and B cells (increase in IgM, IgG). G1-4A is an example of a well-characterized immunomodulator from an Ayurvedic herbal plant obtained by activity-based purification. Both *in vitro* and *in vivo* systems were used to investigate the signaling events. G1-4A protected mice from LPS-induced endotoxin shock by diminishing release of TNFα and IL-1 and increased B cell proliferation (CD19+ and CD69+, CD14+ macrophages, CD11b+ dendritic cells), as shown by increased tritiated thymidine uptake and decreased apoptosis [Figure 3].

Ganguly and Sainis (2001) demonstrated inhibition of cellular immune response by *Tylophora indica* in an experimental mouse model. *Tylophora* extracts inhibit mast cell degranulation, and suppress the early response phase of delayed type hypersensitivity (DTH). In mice, 3.6 mg/kg *Tytophora* suppresses contact sensitivity.[11,12]

Mungantiwar and Sainis (1999) gave 25-100 mg/kg oral *Punarnava (Boerrhavia diffusa*) to mice for 10 days around immunization and showed a significant decrease in DTH. Pre-immunization administration of *Punarnava* had no effect. Interestingly, in vitro tests showed no effect of Punarnava. This suggested a metabolic conversion of the plant alkaloid to its active form *in vivo*. Such discrepancies between in vitro and *in vivo* testing should be always kept in mind in herbal drug research.[19]

Ginger extracts have been shown to have anti-inflammatory effects in a randomized, double-blind, placebo-controlled trial in osteoarthritis of the knee.[14] Also, 63% experienced reduction in knee pain on standing versus 50% in placebo group.[16]

In a limited study, ginger was also found to be useful in relieving pain and swelling in joints of a severe rheumatoid arthritis patient. Similar to curcumin, ginger also inhibits NF-kB via degradation of IKBα, thereby decreasing activation of cyclooxygenase (COX), lipooxygenase (LOX), prostaglandin E2 (PGE2) and Leukotriene B4 (LTB4).[5–7]

## BIOLOGICAL ACTIVITY STUDIES ON CURCUMIN

In recent years, many articles have appeared on curcumin as an anti-inflammatory, antioxidant and anticancer agent, the molecular basis of which was shown to be related to the NF-kB pathway. Curcumin’s anti-inflammatory activity has long been recognized.[20–22] As an antioxidant, curcumin acts by chain-breaking and scavenging free radicals.

Nagabhushan and Bhide (1992) showed curcumin’s activity to inhibit cancer, though its anticancer potential was recognized earlier.[23,24,29] Subsequently, it was confirmed in several studies.[25,26,28,30] A role in the chemoprevention of colon cancer has been suggested.[27,31,32,33]

## *IN VITRO* ASSAY WITH HUMAN SYNOVIOCYTES

Synoviocytes obtained during primary knee replacement of osteoarthritis patients can expand easily in tissue culture over several passages. *In vitro*, they respond to a wide variety of stimuli which activate the NF-kB pathway producing IL-1β, TNFα, IL-6, COX-2, chemokines, PGE2, MMPa and MMP inhibitors, leading to inflammation. Extracts of ginger (*Zingiberis officinale*) and *Alpinia galanga* have been shown to inhibit NF-kB activation. Shakibaei *et al*, (2007) showed that suppression of NF-kB activation by curcumin leads to inhibition of IL-6, and expression of COX2 and MMP-9 in human articular chondrocytes.[13]

This model should be used to study “Jwaraghna”, Shothagna” and “Shofaghna” drugs described in Ayurveda.

## NUTRITION AND IMMUNE RESPONSE

**Figure 5:**
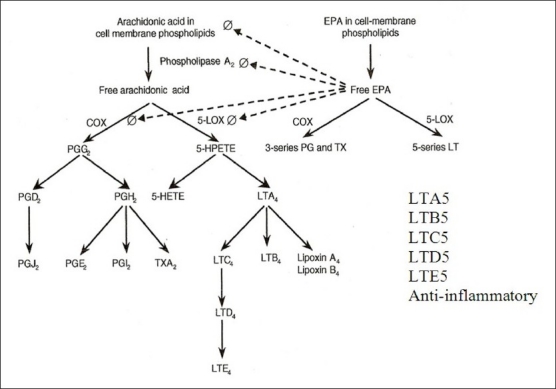
Beneficial effects of EPA/ DHA in cells membrane phospholipids

Calder (2000) has given an excellent review of inflammation in health and disease. He has emphasized the important role of dietary omega-3 polyunsaturated fatty acids-eicosapentaenoic acid (PUFA-EPA) and docosahexaenoic acid (DHA) in the suppression of proinflammatory cytokines, and the need and scope for dietary modification of inflammation [Figure 4]. Increased EPA/DHA in cell membrane phospholipids reduced production of prostanoid (PGI2, TXA2, PGD2, PGE2, PGF2α) while increasing the production of prostacyclin and TXA3, which inhibit platelet aggregation and inflammation [Figure 5].[34]

Zinc, selenium, vitamins A, B6, B12, C, E, and folic acid are important nutrients whose deficiency affects host immune response and thus susceptibility to infection. The important role of vitamin D has only recently been appreciated. Macrophages have receptors for vitamin D, and vitamin D deficiency and vitamin D receptor polymorphism increase susceptibility to tuberculosis.[35]

There is a two-way interaction between nutrients and human genes.[37] How genetic variations influence response to nutrients, and how nutrients influence gene expression, transcription and metabolism are the subject of Nutrigenomics. The effect of maternal malnutrition on fetal insulin-IRS-PI3K AKT pathway is well known to be a basis for metabolic syndrome.

## CHEMOPREVENTION: THE FUTURE APPROACH IN MEDICINE

Cancer chemoprevention by dietary phytochemicals is already an important field of study.[36] Similar chemoprevention of infection (tuberculosis, viral infections including HIV), malignancy and neurodegenerative disorders (Alzheimer’s disease, Parkinson’s disease, etc.) is going to be a major focus in future Ayurvedic drug research.

Through the above studies and other work, the potential of Ayurveda is slowly becoming better appreciated. Recently, a comprehensive review was published by Patwardhan and Mashelkar in the prestigious biomedical journal *Drug Discovery Today*.[38] Ayurveda, however, has its own contributions to offer, based on its own unique approaches to health and healthcare. The dietary studies mentioned in the last two sections begin to offer scientific justification for Ayurveda’s emphasis on *Ahar*, diet, as centrally important to both preventing and curing disease. Further study of *Ahar’s* role in prevention may even obviate the need for curative drugs, and take us beyond both pharmacology and reverse pharmacology.

## RECOMMENDED READING

- RBI Hand book of Receptor Classification and signal transduction 2006 Ed. J. Watling.
- R. D. Lele: Ayurveda and Modern Medicine (2nd ed. 2001) Bharatiya Vidya Bhavan
- R. D. Lele: Clinical Science and Clinical Research, 2008 National Book Depot
